# Supplementary figures and images for: Association between early viral LRTI and subsequent wheezing development, a meta-analysis and sensitivity analyses for studies comparable for confounding factors
Source: PLoS One. 2021 Apr 15;16(4):e0249831. doi: 10.1371/journal.pone.0249831 (PMC8049235; doi:10.1371/journal.pone.0249831)

S1 Fig. Funnel plot for publication for wheezing in children with and without LRTI in infancy

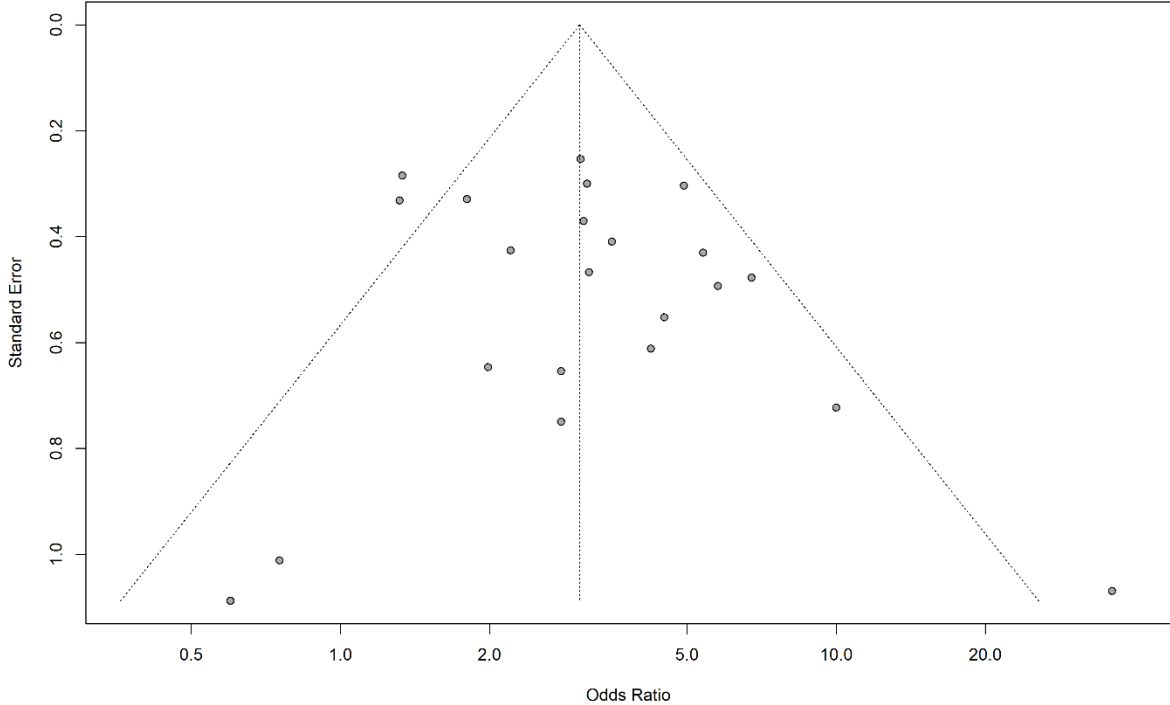

Supplement: S1 Fig — (PDF) [file pone.0249831.s001.pdf]
