# Supplementary material for: Association between early viral LRTI and subsequent wheezing development, a meta-analysis and sensitivity analyses for studies comparable for confounding factors
Source: PLoS One. 2021 Apr 15;16(4):e0249831. doi: 10.1371/journal.pone.0249831 (PMC8049235; doi:10.1371/journal.pone.0249831)
Supplement: S2 Table — (PDF) [file pone.0249831.s003.pdf]

S2 Table. Search strategy in Medline (Pubmed)

**Search**	**Field**	**Search terms**
#1	Respiratory viruses	HRSV OR RSV OR "human respiratory syncytial virus"OR "respiratory syncytial virus" OR HRSV-A OR HRSV-B OR HMPV OR MPV OR "human metapneumovirus" OR metapneumovirus OR HMPV-A OR HMPV-B OR HAdV OR AdV OR Adenovirus OR Adenovirus Infections, Human OR "Human adenovirus" OR HADV-A OR HADV-B OR HADV-C OR HADV-D OR HADV-E OR HADV-F OR HADV-G OR HBoV OR BoV OR Bocavirus OR Bocavirus Infections, Human OR "Human Bocavirus" OR HCoV OR CoV OR Coronavirus OR Coronavirus Infections, Human OR "Human Coronavirus" OR 229E OR OC43 OR NL63 OR HKU1 OR HCoV-229E OR HCoV-OC43 OR HCoV-NL63 OR HCoV-HKU1 OR HPIV OR PIV OR Parainfluenzavirus OR Parainfluenzavirus Infections, Human OR "Human Parainfluenzavirus" OR PIV-1 OR PIV-2 OR PIV-3 OR PIV-4 OR HPIV-1 OR HPIV-2 OR HPIV-3 OR HPIV-4 OR HEV OR EV OR Enterovirus OR Enterovirus Infections, Human OR "Human Enterovirus" OR HRV OR RV OR Rhinovirus OR Rhinoviruses OR Rhinovirus Infections, Human OR "Human Rhinovirus" OR RV-A OR RV-B OR RV-C OR Influenza OR Inf OR "Influenza virus" OR Influenza, Human OR "Influenza-A virus" OR "Influenza-B virus" OR "Influenza-C virus"
#2	LRTI	bronchiolitis OR "severe acute respiratory infections" OR "severe acute respiratory illness" OR ALRI OR "Acute Lower Respiratory Infections" OR "acute lower respiratory tract infections" OR LRTI OR "Lower respiratory tract infections" OR ALRTI OR Croup
#3	Wheezing	wheezing OR "recurrent wheezing" OR "first wheezing" OR "first wheezing episode" OR "subsequent wheezing" OR "wheezing illnesses" OR wheeze OR "persistent recurring wheezing" OR "subsequent recurrent wheezing" OR "wheezing episode" OR "late wheezing" OR "acute wheezing" OR "early wheezing" OR "acute expiratory wheezing" OR "wheezy bronchitis" OR "wheeze-associated RTI" OR "wheezing episodes"
#7		#1 AND #2 AND #3
