# Supplementary material for: Association between early viral LRTI and subsequent wheezing development, a meta-analysis and sensitivity analyses for studies comparable for confounding factors
Source: PLoS One. 2021 Apr 15;16(4):e0249831. doi: 10.1371/journal.pone.0249831 (PMC8049235; doi:10.1371/journal.pone.0249831)
Supplement: S3 Table — (PDF) [file pone.0249831.s004.pdf]

S3 Table. Items for risk of bias assessment

**Newcastle – Ottawa Scale for case cohort studies**	**One star (1)/ No star (0)**
**Selection**	
1) Representativeness of the LRTI cohort	**1**
2) Selection of the non LRTI cohort	**1**
3) Ascertainment of LRTI exposition	**1**
4) Demonstration that wheezing was not present at start of study	**1**
**Comparability**	
1) Comparability of cohorts on the basis of the design or analysis	**2**
**Outcome**	
1) Assessment of wheezing	**1**
2) Was follow-up long enough for outcomes to occur	**1**
3) Adequacy of follow up of cohorts	**1**
Total score	**9**
**Interpretation of the two risk of bias tools**<br><br>• 6-9: Low risk of bias<br>• 0-5: High risk of bias	
