# Supplementary material for: Association between early viral LRTI and subsequent wheezing development, a meta-analysis and sensitivity analyses for studies comparable for confounding factors
Source: PLoS One. 2021 Apr 15;16(4):e0249831. doi: 10.1371/journal.pone.0249831 (PMC8049235; doi:10.1371/journal.pone.0249831)
Supplement: S4 Table — (PDF) [file pone.0249831.s005.pdf]

S4 Table. Main reasons of exclusion of eligible studies

N°	Author, Date	Title	Reason of exclusion
1	Al-Shawwa, 2006	Respiratory syncytial virus bronchiolitis and risk of subsequent wheezing: a matter of severity	No LRTI - group
2	Bacherier, 2012	Determinants of asthma after severe respiratory syncytial virus bronchiolitis.	No LRTI - group
3	Backman, 2018	Asthma and lung function in adulthood after a viral wheezing episode in early childhood.	No LRTI + group
4	Balekian, 2017	Cohort Study of Severe Bronchiolitis during Infancy and Risk of Asthma by Age 5 Years.	No LRTI - group
5	Bergroth, 2016	Post-bronchiolitis use of asthma medication: a prospective 1-year follow-up study	No LRTI - group
6	Bergroth, 2020	Rhinovirus Type in Severe Bronchiolitis and the Development of Asthma.	No LRTI + group
7	Blanken, 2013	Respiratory Syncytial Virus and Recurrent Wheeze in Healthy Preterm Infants.	No LRTI - group
8	Bochkov, 2020	A 14-year Prospective Study of Human Coronavirus Infections in Hospitalized Children: Comparison With Other Respiratory Viruses.	No LRTI - group
9	Bonnelykke, 2015	Association between respiratory infections in early life and later asthma is independent of virus type.	No LRTI - group
10	Bont, 2000	Long-term consequences of respiratory syncytial virus (RSV) bronchiolitis.	Review
11	Bont, 2004	Seasonality of long term wheezing following respiratory syncytial virus lower respiratory tract infection.	No LRTI - group
12	Bosis, 2008	Role of respiratory pathogens in infants hospitalized for a first episode of wheezing and their impact on recurrences	No LRTI + group
13	Bradley, 2005	Severity of respiratory syncytial virus bronchiolitis is affected by cigarette smoke exposure and atopy.	No LRTI + group
14	Carbonell-Estrany, 2015	Long-term burden and respiratory effects of respiratory syncytial virus hospitalization in preterm infants - the SPRING study	Only children with comorbidities recruted
15	Carlsen, 2020	Azithromycin administered for acute bronchiolitis may have a protective effect on subsequent wheezing.	No LRTI - group
16	Chung, 2002	RANTES may be predictive of later recurrent wheezing after respiratory syncytial virus bronchiolitis in infants.	No data on outcomes
17	Cifuentes , 2003	Risk factors for recurrent wheezing following acute bronchiolitis: a 12-month follow-up.	No LRTI - group
18	Coverstone, 2018	Recurrent wheezing in children following human metapneumovirus infection.	Not possible to collect data of interest
19	Del Rosal, 2016	Recurrent wheezing and asthma after bocavirus bronchiolitis.	No LRTI - group
20	Ding, 2019	Comparison of clinical features of acute lower respiratory tract infections in infants with RSV/HRV infection, and incidences of subsequent wheezing or asthma in childhood.	No LRTI - group
21	Dumas, 2019	Severe bronchiolitis profiles and risk of recurrent wheeze by age 3 years.	No LRTI - group
22	Ericksson, 2000	Wheezing following lower respiratory tract infections with respiratory syncytial virus and influenza A in infancy.	No LRTI - group
23	Ermers, 2011	IL-13 genetic polymorphism identifies children with late wheezing after respiratory syncytial virus infection.	No LRTI - group
24	Ermes, 2011	IL10 family member genes IL19 and IL20 are associated with recurrent wheeze after respiratory syncytial virus bronchiolitis.	No LRTI - group

N°	Author, Date	Title	Reason of exclusion
25	Escobar, 2013	Persistent recurring wheezing in the fifth year of life after laboratory-confirmed, medically attended respiratory syncytial virus infection in infancy.	No LRTI - group
26	Everard, 2006	The relationship between respiratory syncytial virus infections and the development of wheezing and asthma in children.	Review
27	Fauroux, 2014	Respiratory morbidity of preterm infants of less than 33 weeks gestation without bronchopulmonary dysplasia: a 12-month follow-up of the CASTOR study cohort.	Not viral laboratory confirmed LRTI
28	Feyzullah, 2014	Effects of wheezing in early childhood in the development of allergic rhinitis in later years.	No LRTI + group
29	Gaffin, 2011	The effect of prophylactic palivizumab on recurrent wheezing in children with an atopic family history.	Editorial
30	Goetghebuer, 2004	Genetic predisposition to wheeze following respiratory syncytial virus bronchiolitis.	No LRTI - group
31	Gómez, 2004	Respiratory repercussions in adults with a history of infantile bronchiolitis	Not viral laboratory confirmed LRTI
32	Guilbert, 2011	Decreased lung function after preschool wheezing rhinovirus illnesses in children at risk to develop asthma.	No LRTI - group
33	Hall, 1984	Long-term prospective study in children after respiratory syncytial virus infection.	No data on outcomes
34	Hasegawa, 2019	Association of Rhinovirus C Bronchiolitis and Immunoglobulin E Sensitization During Infancy With Development of Recurrent Wheeze.	No LRTI - group
35	Hyvarinen, 2005	Teenage asthma after severe early childhood wheezing: An 11-year prospective follow-up.	No LRTI - group
36	Hyvãrinen, 2005	Teenage asthma after severe infantile bronchiolitis or pneumonia.	No data on outcomes
37	Illi, 2001	Early childhood infectious diseases and the development of asthma up to school age: a birth cohort study.	No LRTI - group
38	Jackson, 2008	Wheezing rhinovirus illnesses in early life predict asthma development in high-risk children.	Not possible to collect data of interest
39	Jain, 1974	Acute bronchiolotis and subsequent wheezing.	No abstract and full text available
40	Jedrychowski, 2010	Early wheezing phenotypes and cognitive development of 3-yr-olds. Community-recruited birth cohort study.	No LRTI + group
41	Karaman, 2011	Recurrence of wheezing episodes in children with respiratory syncytial virus and non-respiratory syncytial virus bronchiolitis	No LRTI - group
42	Koponen, 2012	Preschool asthma after bronchiolitis in infancy.	No LRTI - group
43	Korppi, 2004	Respiratory morbidity 20 years after RSV infection in infancy	No data on outcomes
44	Korppi, 2017	IL-10 gene polymorphism is associated with preschool atopy and early-life recurrent wheezing after bronchiolitis in infancy.	No LRTI - group
45	Korsten, 2019	RSV hospitalization in infancy increases the risk of current wheeze at age 6 in late preterm born children without atopic predisposition.	Only children with comorbidities recruted
46	Kotaniemi-syrjänen, 2002	Wheezing requiring hospitalization in early childhood: Predictive factors for asthma in a six-year follow-up.	No data on outcomes
47	Kotaniemi-syrjanen, 2005	Respiratory syncytial virus infection in children hospitalized for wheezing: Virus-specific studies from infancy to preschool years.	No data on outcomes

N°	Author, Date	Title	Reason of exclusion
48	Kusel, 2007	Early-life respiratory viral infections, atopic sensitization, and risk of subsequent development of persistent asthma.	No LRTI - group
49	Lee, 2007	Relationship of early childhood viral exposures to respiratory symptoms, onset of possible asthma and atopy in high risk children: The Canadian asthma primary prevention study	No LRTI - group
50	Lemanske, 2005	Rhinovirus illnesses during infancy predict subsequent childhood wheezing.	Not possible to collect data of interest
51	Lin, 2001	Risk factors of wheeze and allergy after lower respiratory tract infections during early childhood.	No abstract and full text available
52	Liu, 2020	Association between respiratory syncytial virus hospitalization in infancy and childhood asthma.	No data on outcomes
53	Lu, 2016	Predictors of asthma following severe respiratory syncytial virus (RSV) bronchiolitis in early childhood.	No LRTI - group
54	Lukkarinen, 2017	Rhinovirus-induced first wheezing episode predicts atopic but not nonatopic asthma at school age	No LRTI - group
55	Mansbach, 2020	Detection of respiratory syncytial virus or rhinovirus weeks after hospitalization for bronchiolitis and the risk of recurrent wheezing.	No LRTI - group
56	Marlow, 2019	Assessing the association between bronchiolitis in infancy and recurrent wheeze: a whole English birth cohort case-control study.	Not viral laboratory confirmed LRTI
57	Martinez, 1998	Differential immune responses to acute lower respiratory illness in early life and subsequent development of persistent wheezing and asthma.	No LRTI - group
58	McConnochie, 1984	Bronchiolitis as a possible cause of wheezing in childhood: new evidence	Not viral laboratory confirmed LRTI
59	McConnochie, 1985	Predicting clinically significant lower respiratory tract illness in childhood following mild bronchiolitis	Not viral laboratory confirmed LRTI
60	McConnochie, 1989	Wheezing at 8 and 13 years: changing importance of bronchiolitis and passive smoking	Not viral laboratory confirmed LRTI
61	Mejias, 2020	Risk of childhood wheeze and asthma after respiratory syncytial virus infection in full-term infants.	No LTRI + group
62	Midulla, 2012	Rhinovirus bronchiolitis and recurrent wheezing: 1-year follow-up.	Not viral laboratory confirmed LRTI
63	Midulla, 2014	Recurrent wheezing 36 months after bronchiolitis is associated with rhinovirus infections and blood eosinophilia.	No LRTI - group
64	Mikalsen, 2012	The outcome after severe bronchiolitis is related to gender and virus	No data on outcomes
65	Mok, 1982	Outcome of acute lower respiratory tract infection in infants: preliminary report of seven-year follow-up study	Not viral laboratory confirmed LRTI
66	Mok, 1984	Outcome for acute bronchitis, bronchiolitis, and pneumonia in infancy	Not viral laboratory confirmed LRTI

N°	Author, Date	Title	Reason of exclusion
67	Murray, 1992	Respiratory status and allergy after bronchiolitis	Not viral laboratory confirmed LRTI
68	Narita, 2011	Relationship between lower respiratory tract infections caused by respiratory syncytial virus and subsequent development of asthma in Japanese children.	No LRTI - group
69	Nenna, 2015	Viral Load in Infants Hospitalized for Respiratory Syncytial Virus Bronchiolitis Correlates with Recurrent Wheezing at Thirty-Six-Month Follow-Up.	No LRTI - group
70	Nicolai, 2017	Risk Factors for Virus-induced Acute Respiratory Tract Infections in Children Younger Than 3 Years and Recurrent Wheezing at 36 Months Follow-Up After Discharge.	No LRTI - group
71	Noble, 1997	Respiratory status and allergy nine to 10 years after acute bronchiolitis	Not viral laboratory confirmed LRTI
72	O'Callaghan-Gordo, 2013	Lower respiratory tract infections associated with rhinovirus during infancy and increased risk of wheezing during childhood. A cohort study.	No LRTI - group
73	Oymar, 2001	Eosinophil counts and urinary eosinophil protein X in children hospitalized for wheezing during the first year of life: prediction of recurrent wheezing.	No LRTI - group
74	Palmer, 2011	Respiratory outcomes, utilization and costs 12 months following a respiratory syncytial virus diagnosis among commercially insured late-preterm infants	Only children with comorbidities recruted
75	Panitch, 2007	The relationship between early respiratory viral infections and subsequent wheezing and asthma.	Review
76	Petrarca, 2018	Acute bronchiolitis: Influence of viral co-infection in infants hospitalized over 12 consecutive epidemic seasons.	No LRTI - group
77	Pifferi, 2001	Eosinophil cationic protein in infants with respiratory syncytial virus bronchiolitis: predictive value for subsequent development of persistent wheezing.	No LRTI - group
78	Piippo-Savolainen, 2004	Asthma and Lung Function 20 Years After Wheezing in Infancy: Results From a Prospective Follow-up Study	Not viral laboratory confirmed LRTI
79	Piippo-Savolainen, 2006	Early predictors for adult asthma and lung function abnormalities in infants hospitalized for bronchiolitis: A prospective 18-to 20-year follow-up.	No LRTI - group
80	Piippo-Savolainen, 2007	Adult asthma after non-respiratory syncytial virus bronchiolitis in infancy: Subgroup analysis of the 20-year prospective follow-up study.	No LRTI - group
81	Piippo-Savolainen, 2007	Does blood eosinophilia in wheezing infants predict later asthma? A prospective 18-20-year follow-up.	No LRTI - group
82	Ramilo, 2018	Respiratory Syncytial Virus-induced Acute Disease Severity and Long-Term Wheezing Uncovering the Unexpected.	Editorial
83	Ramsey, 2007	Respiratory illnesses in early life and asthma and atopy in childhood.	No LRTI - group
84	Reijonen, 2000	Predictors of asthma three years after hospital admission for wheezing in infancy.	No LRTI - group
85	Rinawi, 2017	Bronchiolitis in young infants: is it a risk factor for recurrent wheezing in childhood?	Not viral laboratory confirmed LRTI

N°	Author, Date	Title	Reason of exclusion
86	Romero, 2010	Serious early childhood wheezing after respiratory syncytial virus lower respiratory tract illness in preterm infants	Only children with comorbidities recruted
87	Rubner, 2017	Early life rhinovirus wheezing, allergic sensitization, and asthma risk at adolescence.	No LRTI - group
88	Ruotsalainen, 2010	Adulthood asthma after wheezing in infancy: a questionnaire study at 27 years of age	Not viral laboratory confirmed LRTI
89	Ruotsalainen, 2013	Adolescent asthma after rhinovirus and respiratory syncytial virus bronchiolitis	Not viral laboratory confirmed LRTI
90	Ruotsalainen, 2013	No association between overweight and asthma or allergy in adolescence after wheezing in infancy	Not viral laboratory confirmed LRTI
91	Santos, 2011	Pneumonia in the first 2 years of life, and asthma in preschool-age children.	Not viral laboratory confirmed LRTI
92	Schauer, 2002	RSV bronchiolitis and risk of wheeze and allergic sensitisation in the first year of life.	Follow up duration < 1 year
93	Sigurs, 1994	Eosinophil cationic protein in nasal secretion and in serum and myeloperoxidase in serum in respiratory syncytial virus bronchiolitis: relation to asthma and atopy	No LRTI - group
94	Sigurs, 2004	Does bronchiolitis caused by RSV predispose to atopic asthma?	Review
95	Sigurs, 2007	Respiratory Syncytial Virus lower respiratory tract illness in infancy and subsequent morbidity.	Comment on an article
96	Simões, 2010	The effect of respiratory syncytial virus on subsequent recurrent wheezing in atopic and nonatopic children.	Only children with comorbidities recruted
97	Sims, 1981	Atopy does not predispose to RSV bronchiolitis or postbronchiolitic wheezing.	No LRTI - group
98	Skirrow, 2019	Preschool respiratory hospital admissions following infant bronchiolitis: a birth cohort study.	No data on outcomes
99	Sly, 1989	Childhood asthma following hospitalisation with acute viral bronchiolitis in infancy	No LRTI - group
100	Stein, 1999	Respiratory syncytial virus in early life and risk of wheeze and allergy by age 13 years.	Not possible to collect data of interest
101	Stein, 2009	Long-term airway morbidity following viral LRTI in early infancy: recurrent wheezing or asthma?	Review
102	Stensballe, 2009	The causal direction in the association between respiratory syncytial virus hospitalization and asthma.	No data on outcomes
103	Szabo, 2014	A Population-Based Study of Childhood Respiratory Morbidity after Severe Lower Respiratory Tract Infections in Early Childhood.	No data on outcomes
104	Takeyama, 2014	Clinical and epidemiologic factors related to subsequent wheezing after virus-induced lower respiratory tract infections in hospitalized pediatric patients younger than 3 years.	No LRTI - group

N°	Author, Date	Title	Reason of exclusion
105	Teeratakulpisarn, 2014	Rhinovirus infection in children hospitalized with acute bronchiolitis and its impact on subsequent wheezing or asthma: a comparison of etiologies.	No LRTI - group
106	Tian, 2009	[The correlation factor about respiratory syncytial virus bronchiolitis and post-bronchiolitis wheezing in infant].	No LRTI - group
107	Toivonen, 2019	Acute respiratory infections in early childhood and risk of asthma at age 7 years.	No LRTI - group
108	Törmänen, 2018	Risk factors for asthma after infant bronchiolitis	Not viral laboratory confirmed LRTI
109	Turner, 2002	Reduced lung function both before bronchiolitis and at 11 years	Not viral laboratory confirmed LRTI
110	Turunen, 2017	Clinical and Virus Surveillance After the First Wheezing Episode Special Reference to Rhinovirus A and C Species.	No LRTI - group
111	Valkonen, 2009	Recurrent wheezing after respiratory syncytial virus or non-respiratory syncytial virus bronchiolitis in infancy: a 3-year follow-up.	No LRTI - group
112	van der Sande, 2002	Severe respiratory syncytial virus infection in early life is associated with increased type 2 cytokine production in Gambian children.	Not possible to collect data of interest
113	van Meel, 2018	A population-based prospective cohort study examining the influence of early-life respiratory tract infections on school-age lung function and asthma.	No LRTI - group
114	van Meel, 2020	Airway bacterial carriage and childhood respiratory health: A population-based prospective cohort study.	Not viral laboratory confirmed LRTI
115	Weber, 1999	Respiratory illness after severe respiratory syncytial virus disease in infancy in The Gambia.	Not possible to collect data of interest
116	Welliver, 1986	Predictive value of respiratory syncytial virus-specific IgE responses for recurrent wheezing following bronchiolitis.	No LRTI - group
117	Welliver, 1993	The relationship of RSV-specific immunoglobulin E antibody responses in infancy, recurrent wheezing, and pulmonary function at age 7-8 years.	No LRTI - group
118	Wennergren, 1997	Wheezing bronchitis reinvestigated at the age of 10 years	No LRTI - group
119	Zhang, 2020	Airway microbiome, host immune response and recurrent wheezing in infants with severe respiratory syncytial virus bronchiolitis.	No LRTI - group
120	Zhou, 2016	Azithromycin therapy during respiratory syncytial virus bronchiolitis: Upper airway microbiome alterations and subsequent recurrent wheeze.	No LRTI - group
121	Zomer-Kooijker, 2014	Decreased lung function precedes severe respiratory syncytial virus infection and post-respiratory syncytial virus wheeze in term infants.	No LRTI - group
