# Supplementary material for: Association between early viral LRTI and subsequent wheezing development, a meta-analysis and sensitivity analyses for studies comparable for confounding factors
Source: PLoS One. 2021 Apr 15;16(4):e0249831. doi: 10.1371/journal.pone.0249831 (PMC8049235; doi:10.1371/journal.pone.0249831)
Supplement: S5 Table — (PDF) [file pone.0249831.s006.pdf]

S5 Table. Baseline Characteristics of Studies Meeting Inclusion Criteria

**Characteristics**	**N = 22**	**%**
**Year of publication**	1978-2017	
**Period of LRTI**	1960-2005	
**Follow-up duration (Years)**	1,5-10	
**Sampling**		
Non probabilistic	21	95.5
Probabilistic	1	4.6
**Timing of exposure collection**		
Prospetively	18	77.3
Retrospectively	4	9.1
**Country**		
Australia	1	4.6
Chile	1	4.6
Danemark	2	9.1
Danmark	1	4.6
England	1	4.6
Finland	1	4.6
Netherland	1	4.6
Norway	1	4.6
Qatar	1	4.6
Russia	1	4.6
Spain	2	9.1
Sweden	6	27.3
UK	2	9.1
United Kingdom	1	4.6
**UNSD Region**		

**Characteristics**	**N = 22**	**%**
Eastern Europe	1	4.6
Northern Europe	15	68.2
Oceania	1	4.6
South America	1	4.6
Southern Europe	2	9.1
Western Asia	1	4.6
Western Europe	1	4.6
**Country income level**		
High-income economies	21	95.5
Upper-middle-income economies	1	4.6
**Age range at recrutment**		
< 6 months	5	22.7
< 1 year	13	59.1
< 2 years	3	13.7
Unclear/Not reported	1	4.6
**Age range at interview**		
2-5 years	9	40.9
5-10 years	8	36.3
10-15 years	1	4.5
15-20 years	1	4.5
Unclear/Not reported	3	13.6
**Hospitalization LRTI +**		
Hospitalized	22	100.0
**Hospitalization LRTI -**		
Ambulatory	8	36.4
Hospitalized	7	31.8

**Characteristics**	**N = 22**	**%**
Hospitalized/ambulatory	2	9.1
Unclear/Not reported	5	22.7
**First episode of LRTI**		
No	3	13.6
Yes	15	68.2
Unclear/Not reported	4	18.2
**Virus screened for LRTI +**		
Adenovirus type 7	1	4.6
Human Metapneumovirus (hMPV)	1	4.6
Human Respiratory Syncytial Virus (HRSV)	20	90.9
**Sample types**		
Nasopharyngeal	9	40.9
Unclear/ Not reported	13	59.1
**Detection assays**		
Classical RT-PCR	1	4.6
Classical RT-PCR, ELISA	2	9.1
Culture	1	4.6
Culture, Rapid antigen	1	4.6
ELISA, latex agglutination test	1	4.6
Enzyme immunoassay	3	13.6
Immunoassay method	1	4.6
Immunoflorescent assay	2	9.1
Immunoflorescent assay, enzyme immunoassay	2	9.1
Immunofluorescence, Culture	2	9.1
Immunofluorescence, RT-PCR	2	9.1
Unclear/Not reported	4	18.2

**Characteristics**	**N = 22**	**%**
**Type of LRTI**		
Bronchiolitis	15	68.2
LRTI not specified	6	27.3
Pneumonia	1	4.6
**Type of wheezing**		
Any wheezing	2	9.1
Current wheezing	6	27.3
Recurrent wheezing	13	59.1
Wheezing in the last 12 months	1	4.6

LRTI: Lower Respiratory Tract Infections; HRSV: Human respiratory syncytial virus; HAdV: Human adenovirus; HMPV: Human metapneumovirus
