# Supplementary material for: Association between early viral LRTI and subsequent wheezing development, a meta-analysis and sensitivity analyses for studies comparable for confounding factors
Source: PLoS One. 2021 Apr 15;16(4):e0249831. doi: 10.1371/journal.pone.0249831 (PMC8049235; doi:10.1371/journal.pone.0249831)
Supplement: S6 Table — (PDF) [file pone.0249831.s007.pdf]

S6 Table: Risk of bias assessment

Author, Year	Representativeness of the LRTI +	Selection of the LRTI -	Ascertainment of LRTI	Demonstration that wheezing was not present at start of study	Comparability of cohorts on the basis of the design or analysis	Assessment of wheezing	Was follow-up long enough for wheezing to occur	Adequacy of follow up of cohorts	Total score	Risk of bias
Bertrand, 2015	1	1	1	0	2	1	1	1	8	Low risk of bias
Fjaerli, 2005	1	1	1	0	2	1	1	1	8	Low risk of bias
García-García, 2007, HMPV	1	1	1	1	0	1	1	1	7	Low risk of bias
García-García, 2007, HRSV	1	1	1	1	0	1	1	1	7	Low risk of bias
Henderson, 2005, 30–42 months	1	0	1	1	0	1	1	1	6	Low risk of bias
Henderson, 2005, 69–81 months	1	0	1	1	0	1	1	1	6	Low risk of bias
Juntti, 2003	1	1	1	0	2	1	1	1	8	Low risk of bias
Kristjánsson, 2006	1	0	1	0	2	0	1	1	6	Low risk of bias
Osundwa, 1993	1	1	1	0	1	1	1	1	7	Low risk of bias
Poorisrisak, 2010	1	0	1	1	0	1	1	1	6	Low risk of bias
Pullan, 1982	1	0	1	1	0	1	1	1	6	Low risk of bias
Sigurs, 1995, 1 year	1	1	1	1	1	1	1	1	8	Low risk of bias
Sigurs, 1995, 3 years	1	1	1	1	1	1	1	1	8	Low risk of bias
Sigurs, 2000	1	1	1	1	2	1	1	1	9	Low risk of bias
Sigurs, 2005	1	1	1	1	2	1	1	1	9	Low risk of bias
Sigurs, 2010	1	1	1	1	2	1	1	1	9	Low risk of bias
Sims, 1978	1	1	1	1	1	1	1	1	8	Low risk of bias
Singleton, 2003	1	1	1	1	1	1	1	1	8	Low risk of bias
Sly, 1984	1	1	1	0	2	1	1	1	8	Low risk of bias
Stensballe, 2017, 1,5 years	1	1	1	1	1	1	1	1	8	Low risk of bias
Stensballe, 2017, 5 years	1	1	1	1	1	1	1	1	8	Low risk of bias
Zomer-Kooijker, 2014	1	1	1	1	1	1	1	1	8	Low risk of bias
