# Supplementary material for: Association between early viral LRTI and subsequent wheezing development, a meta-analysis and sensitivity analyses for studies comparable for confounding factors
Source: PLoS One. 2021 Apr 15;16(4):e0249831. doi: 10.1371/journal.pone.0249831 (PMC8049235; doi:10.1371/journal.pone.0249831)
Supplement: S8 Table — (PDF) [file pone.0249831.s009.pdf]

S8 Table. P-value of Khi-2 and Fisher exact tests for qualitative confounding factors

		Data extracted from included studies				Results from this study		
Author, year	Qualitative confounding factor	Total number of LRTI +	Number of LRTI + with confounding factors	Total number of LRTI -	Total number of LRTI - with confounding factors	P-value Khi-2 test	P-value Fisher exact test	Status
Bertrand, 2015	Atopy in parents	14	7	5	2	1	1	Symmetric
Bertrand, 2015	Male gender	14	6	5	3	0,891	0,628	Symmetric
Fjaerli, 2005	Current asthma	35	12	64	12	0,139	0,093	Symmetric
Fjaerli, 2005	Current atopy	35	16	64	33	0,729	0,675	Symmetric
Fjaerli, 2005	Current eczema	35	14	64	28	0,882	0,832	Symmetric
Fjaerli, 2005	Male gender	35	20	64	36	1	1	Symmetric
Fjaerli, 2005	Parental smoking	35	19	64	28	0,428	0,4	Symmetric
Fjaerli, 2005	Siblings in the house	35	11	64	26	0,492	0,394	Symmetric
García-García, 2007, HMPV	Allergic rhinitis	23	5	30	5	0,91	0,73	Symmetric
García-García, 2007, HMPV	Antiasthmatic treatment	23	7	30	2	0,055	0,031	Asymmetric
García-García, 2007, HMPV	Asthma admissions	23	5	30	0	0,027	0,012	Asymmetric
García-García, 2007, HMPV	Asthma in children	23	16	30	4	0	0	Asymmetric
García-García, 2007, HMPV	Asthma in father	23	3	30	4	1	1	Symmetric
García-García, 2007, HMPV	Asthma in mother	23	6	30	6	0,846	0,743	Symmetric
García-García, 2007, HMPV	Asthma in siblings	23	8	30	4	0,129	0,098	Symmetric
García-García, 2007, HMPV	Atopic dermatitis	23	10	30	11	0,827	0,778	Symmetric
García-García, 2007, HMPV	Atopy in children	23	8	30	6	0,371	0,346	Symmetric
García-García, 2007, HMPV	Atopy in father	23	6	30	5	0,62	0,501	Symmetric
García-García, 2007, HMPV	Atopy in mother	23	7	30	6	0,58	0,522	Symmetric
García-García, 2007, HMPV	Atopy in siblings	23	6	30	3	0,239	0,154	Symmetric
García-García, 2007, HMPV	Current atopy	23	1	30	2	1	1	Symmetric
García-García, 2007, HMPV	Male gender	23	14	30	13	0,323	0,271	Symmetric
García-García, 2007, HMPV	Maternal smoking	23	7	30	12	0,667	0,569	Symmetric
García-García, 2007, HMPV	Paternal smoking	23	8	30	10	1	1	Symmetric
García-García, 2007, HMPV	Pets at home	23	7	30	13	0,5	0,4	Symmetric
García-García, 2007, HMPV	Premature birth	23	9	30	3	0,029	0,019	Asymmetric
García-García, 2007, HRSV	Allergic rhinitis	32	7	30	5	0,844	0,751	Symmetric
García-García, 2007, HRSV	Antiasthmatic treatment	32	10	30	2	0,033	0,023	Asymmetric
García-García, 2007, HRSV	Asthma admissions	32	5	30	0	0,073	0,053	Symmetric
García-García, 2007, HRSV	Asthma in children	32	20	30	4	0	0	Asymmetric
García-García, 2007, HRSV	Asthma in father	32	5	30	4	1	1	Symmetric
García-García, 2007, HRSV	Asthma in mother	32	6	30	6	1	1	Symmetric

García-García, 2007, HRSV	Asthma in siblings	32	5	30	4	1	1	Symmetric
García-García, 2007, HRSV	Atopic dermatitis	32	11	30	11	1	1	Symmetric
García-García, 2007, HRSV	Atopy in children	32	8	30	6	0,868	0,764	Symmetric
García-García, 2007, HRSV	Atopy in father	32	4	30	5	0,917	0,728	Symmetric
García-García, 2007, HRSV	Atopy in mother	32	13	30	6	0,138	0,102	Symmetric
García-García, 2007, HRSV	Atopy in siblings	32	11	30	3	0,047	0,033	Asymmetric
García-García, 2007, HRSV	Current atopy	32	2	30	2	1	1	Symmetric
García-García, 2007, HRSV	Male gender	32	17	30	13	0,605	0,459	Symmetric
García-García, 2007, HRSV	Maternal smoking	32	21	30	12	0,077	0,074	Symmetric
García-García, 2007, HRSV	Paternal smoking	32	18	30	10	0,12	0,081	Symmetric
García-García, 2007, HRSV	Pets at home	32	8	30	13	0,209	0,18	Symmetric
García-García, 2007, HRSV	Premature birth	32	6	30	3	0,537	0,475	Symmetric
Henderson, 2005, 30–42 months	History of atopy	48	7	6377	1319	0,389	0,372	Symmetric
Henderson, 2005, 30–42 months	Asthma in children	73	28	8039	1613	0	0	Asymmetric
Henderson, 2005, 69–81 months	History of atopy	48	7	6377	1319	0,389	0,372	Symmetric
Henderson, 2005, 69–81 months	Asthma in children	73	28	8039	1613	0	0	Asymmetric
Juntti, 2003	Allergic rhinitis	76	7	76	10	0,607	0,608	Symmetric
Juntti, 2003	History of asthma	76	20	76	12	0,164	0,163	Symmetric
Juntti, 2003	History of eczema	76	22	76	24	0,86	0,86	Symmetric
Juntti, 2003	Male gender	76	47	76	47	1	1	Symmetric
Juntti, 2003	Maternal smoking during pregnancy	76	8	76	14	0,249	0,249	Symmetric
Juntti, 2003	Pets at home	76	31	76	41	0,144	0,143	Symmetric
Juntti, 2003	Smoke exposure	76	32	76	25	0,315	0,315	Symmetric
Kristjánsson, 2006	Family history of atopy	17	14	25	20	1	1	Symmetric
Kristjánsson, 2006	Male gender	17	9	27	25	0,007	0,007	Asymmetric
Osundwa, 1993	Male gender	70	42	70	45	0,727	0,728	Symmetric
Poorisrisak, 2010	Asthma in children	74	19	74	16	0,699	0,699	Symmetric
Poorisrisak, 2010	Atopic dermatitis	74	30	74	27	0,735	0,736	Symmetric
Poorisrisak, 2010	Atopy in children	74	6	74	14	0,092	0,091	Symmetric
Pullan, 1982	Antiasthmatic treatment	130	7	111	5	0,987	1	Symmetric
Pullan, 1982	Maternal smoking	130	68	111	40	0,016	0,014	Asymmetric
Pullan, 1982	Paternal smoking	130	66	111	45	0,145	0,121	Symmetric
Sigurs, 1995, 1 year	Asthma in parents	47	0	93	3	0,531	0,551	Symmetric
Sigurs, 1995, 1 year	Atopic dermatitis	47	2	93	3	1	1	Symmetric
Sigurs, 1995, 1 year	Atopy in parents	47	9	93	18	1	1	Symmetric
Sigurs, 1995, 1 year	Heredity for asthma	47	11	93	19	0,852	0,67	Symmetric
Sigurs, 1995, 1 year	Heredity for atopy	47	18	93	34	0,987	0,855	Symmetric
Sigurs, 1995, 1 year	History of pertussis	47	11	93	17	0,623	0,507	Symmetric
Sigurs, 1995, 1 year	Male gender	47	21	93	42	1	1	Symmetric
Sigurs, 1995, 1 year	Pets at home	47	12	93	36	0,173	0,135	Symmetric

Sigurs, 1995, 1 year	Smoke exposure	47	21	93	42	1	1	Symmetric
Sigurs, 1995, 3 years	Asthma in parents	47	0	93	3	0,531	0,551	Symmetric
Sigurs, 1995, 3 years	Atopic dermatitis	47	2	93	3	1	1	Symmetric
Sigurs, 1995, 3 years	Atopy in parents	47	9	93	18	1	1	Symmetric
Sigurs, 1995, 3 years	Heredity for asthma	47	11	93	19	0,852	0,67	Symmetric
Sigurs, 1995, 3 years	Heredity for atopy	47	18	93	34	0,987	0,855	Symmetric
Sigurs, 1995, 3 years	History of pertussis	47	11	93	17	0,623	0,507	Symmetric
Sigurs, 1995, 3 years	Male gender	47	21	93	42	1	1	Symmetric
Sigurs, 1995, 3 years	Pets at home	47	12	93	36	0,173	0,135	Symmetric
Sigurs, 1995, 3 years	Smoke exposure	47	21	93	42	1	1	Symmetric
Sigurs, 2000	Heredity for asthma	47	21	93	27	0,098	0,089	Symmetric
Sigurs, 2000	Heredity for atopy	47	33	93	60	0,628	0,572	Symmetric
Sigurs, 2000	Male gender	47	21	93	42	1	1	Symmetric
Sigurs, 2000	Pets at home	47	22	93	52	0,401	0,371	Symmetric
Sigurs, 2000	Smoke exposure	47	24	93	49	0,998	0,86	Symmetric
Sigurs, 2005	Asthma in parents	47	17	93	25	0,349	0,329	Symmetric
Sigurs, 2005	Atopy in parents	47	28	93	50	0,636	0,59	Symmetric
Sigurs, 2005	Pets at home	47	29	93	68	0,235	0,179	Symmetric
Sigurs, 2005	Smoke exposure	47	16	93	39	0,472	0,464	Symmetric
Sigurs, 2010	Smoke exposure	45	19	92	40	1	1	Symmetric
Sigurs, 2010	Asthma in parents	46	18	92	25	0,217	0,175	Symmetric
Sigurs, 2010	Atopy in parents	46	30	92	52	0,426	0,362	Symmetric
Sigurs, 2010	Pets at home	46	24	92	52	0,762	0,717	Symmetric
Sims, 1978	Male gender	35	17	35	17	1	1	Symmetric
Singleton, 2003	Male gender	95	53	113	56	0,449	0,405	Symmetric
Singleton, 2003	Premature birth	95	13	113	3	0,007	0,004	Asymmetric
Singleton, 2003	Smoke exposure	95	45	113	50	0,756	0,677	Symmetric
Sly, 1984	Atopy in children	20	6	20	11	0,201	0,2	Symmetric
Sly, 1984	Male gender	20	10	20	12	0,751	0,751	Symmetric
Sly, 1984	Smoke exposure	20	12	20	14	0,74	0,741	Symmetric
Stensballe, 2017, 1,5 years	Atopic dermatitis	39	2	23	2	0,986	0,623	Symmetric
Stensballe, 2017, 1,5 years	Atopy in parents	39	15	23	8	0,986	1	Symmetric
Stensballe, 2017, 1,5 years	Male gender	39	24	23	16	0,716	0,591	Symmetric
Stensballe, 2017, 1,5 years	Maternal smoking	39	26	23	14	0,852	0,784	Symmetric
Stensballe, 2017, 1,5 years	Premature birth	39	4	23	3	1	1	Symmetric
Stensballe, 2017, 5 years	Atopic dermatitis	39	2	23	2	0,986	0,623	Symmetric
Stensballe, 2017, 5 years	Atopy in parents	39	15	23	8	0,986	1	Symmetric
Stensballe, 2017, 5 years	Male gender	39	24	23	16	0,716	0,591	Symmetric
Stensballe, 2017, 5 years	Maternal smoking	39	26	23	14	0,852	0,784	Symmetric
Stensballe, 2017, 5 years	Premature birth	39	4	23	3	1	1	Symmetric

Zomer-Kooijker, 2014	Smoke exposure	159	42	451	126	0,79	0,757	Symmetric
Zomer-Kooijker, 2014	Atopy in mother	154	66	466	224	0,303	0,266	Symmetric
Zomer-Kooijker, 2014	Maternal high educational level	153	53	470	301	0	0	Asymmetric
Zomer-Kooijker, 2014	Pets at home	154	79	475	274	0,196	0,191	Symmetric
Zomer-Kooijker, 2014	Breastfeeding	155	97	515	376	0,016	0,016	Asymmetric
Zomer-Kooijker, 2014	Day care attendance	155	52	515	227	0,025	0,02	Asymmetric
Zomer-Kooijker, 2014	Maternal smoking during pregnancy	155	27	548	26	0	0	Asymmetric
Zomer-Kooijker, 2014	Siblings in the house	155	134	548	297	0	0	Asymmetric
Zomer-Kooijker, 2014	Male gender	159	87	549	265	0,18	0,177	Symmetric
