# Supplementary material for: Association between early viral LRTI and subsequent wheezing development, a meta-analysis and sensitivity analyses for studies comparable for confounding factors
Source: PLoS One. 2021 Apr 15;16(4):e0249831. doi: 10.1371/journal.pone.0249831 (PMC8049235; doi:10.1371/journal.pone.0249831)
Supplement: S9 Table — (PDF) [file pone.0249831.s010.pdf]

S8 Table. P-value of Student test for quantitative confounding factors

		Data extracted from included studies						Results from this study	
Author, year	Quantitative confounding factors	Total number of LRTI +	Mean for LRTI +	SD for LRTI +	Total number of LRTI -	Mean for LRTI -	SD for LRTI -	P-Value for Student Test with Unequal Variance	Status
García-García, 2007, HMPV	Age at interview (Years)	23	3,70	0,92	30	3,90	0,61	0,187	Symmetric
García-García, 2007, HRSV	Age at interview (Years)	32	4,03	0,65	30	3,90	0,61	0,21	Symmetric
Sims, 1978	Age at interview (Years)	35	8,32	0,28	35	8,40	0,33	0,139	Symmetric
Sly, 1984	Age at interview (Years)	20	16,10	5,70	20	12,70	3,20	0,013	Asymmetric
Zomer-Kooijker, 2014	Age at interview (Years)	159	5,93	0,55	549	6,08	1,92	0,049	Asymmetric
Osundwa, 1993	Age at recrutment (months)	70	3,80	3,00	70	4,00	3,00	0,347	Symmetric
Sly, 1984	Age at recrutment (months)	20	3,20	2,30	20	5,70	3,40	0,005	Asymmetric
Stensballe, 2017, 1,5 years	Age at recrutment (months)	39	0,68	0,56	23	0,84	0,62	0,164	Symmetric
Stensballe, 2017, 5 years	Age at recrutment (months)	39	0,68	0,56	23	0,84	0,62	0,164	Symmetric
Sigurs, 1995, 3 years	Birth weight (grams)	47	3314,00	556,00	93	3520,00	657,00	0,027	Asymmetric
Sigurs, 1995, 1 year	Birth weight (grams)	47	3314,00	556,00	93	3520,00	657,00	0,027	Asymmetric
Sims, 1978	Birth weight (grams)	35	3220,00	490,00	35	3320,00	460,00	0,191	Symmetric
Stensballe, 2017, 1,5 years	Birth weight (grams)	39	3420,00	507,90	23	3486,67	474,10	0,302	Symmetric
Stensballe, 2017, 5 years	Birth weight (grams)	39	3420,00	507,90	23	3486,67	474,10	0,302	Symmetric
Sigurs, 1995, 3 years	Breastfeeding period (months)	47	4,20	3,30	93	4,80	3,40	0,159	Symmetric
Sigurs, 1995, 1 year	Breastfeeding period (months)	47	4,20	3,30	93	4,80	3,40	0,159	Symmetric
Sigurs, 1995, 3 years	Gestational age (weeks)	47	38,90	2,00	93	39,80	1,60	0,004	Asymmetric
Sigurs, 1995, 1 year	Gestational age (weeks)	47	38,90	2,00	93	39,80	1,60	0,004	Asymmetric
Sigurs, 2000	Height at interview (cm)	47	126,00	6,58	93	127,00	5,22	0,183	Symmetric
Sigurs, 1995, 3 years	Height at interview (cm)	47	97,20	4,00	93	96,80	4,00	0,289	Symmetric
Sigurs, 2010	Height at interview (cm)	46	174,00	10,00	92	174,00	10,00	0,5	Symmetric
Sigurs, 2005	Height at interview (cm)	47	160,30	8,80	93	162,30	8,30	0,1	Symmetric
Sigurs, 1995, 1 year	Height at interview (cm)	47	97,20	4,00	93	96,80	4,00	0,289	Symmetric
Sims, 1978	Height at interview (cm)	35	127,90	63,90	35	126,40	5,25	0,445	Symmetric
Zomer-Kooijker, 2014	Height at interview (cm)	159	118,80	9,90	549	118,10	5,57	0,197	Symmetric
Sims, 1978	Mean age of siblings (Years)	35	10,90	5,13	35	8,50	4,17	0,018	Asymmetric
Sigurs, 2000	Number of siblings	47	1,80	0,97	93	1,78	1,10	0,456	Symmetric
Sigurs, 1995, 3 years	Number of siblings	47	1,30	0,90	93	1,10	1,00	0,117	Symmetric
Sigurs, 2005	Number of siblings	47	2,00	0,95	93	1,80	0,99	0,125	Symmetric
Sigurs, 1995, 1 year	Number of siblings	47	1,30	0,90	93	1,10	1,00	0,117	Symmetric
Sims, 1978	Number of siblings	35	2,10	1,45	35	1,30	0,86	0,003	Asymmetric
Sigurs, 2000	Weight at interview (Kg)	47	27,00	4,63	93	27,10	4,21	0,451	Symmetric
Sigurs, 1995, 3 years	Weight at interview (Kg)	47	15,60	1,80	93	15,40	1,80	0,268	Symmetric
Sigurs, 2010	Weight at interview (Kg)	46	65,00	12,00	92	70,00	13,00	0,014	Asymmetric
Sigurs, 2005	Weight at interview (Kg)	47	52,20	10,60	93	52,90	11,20	0,359	Symmetric
Sigurs, 1995, 1 year	Weight at interview (Kg)	47	15,60	1,80	93	15,40	1,80	0,268	Symmetric
Sims, 1978	Weight at interview (Kg)	35	26,58	4,00	35	26,20	3,95	0,345	Symmetric

Zomer-Kooijker, 2014	Weight at interview (Kg)	159	21,60	4,77	549	21,77	3,20	0,339	Symmetric
