# Supplementary material for: Association between early viral LRTI and subsequent wheezing development, a meta-analysis and sensitivity analyses for studies comparable for confounding factors
Source: PLoS One. 2021 Apr 15;16(4):e0249831. doi: 10.1371/journal.pone.0249831 (PMC8049235; doi:10.1371/journal.pone.0249831)
Supplement: S10 Table — (PDF) [file pone.0249831.s011.pdf]

S10 Table. Subgroup analyses of wheezing in children with LRTI in infancy and control without respiratory diseases.

	OR (95%CI)	95% Prediction interval	N Studies	N LRTI cases	N controls	H (95%CI)	I² (95%CI)	P heterogeneity	P-value Egger test	P-value subgroup difference
**Sampling**										0.505
Non probabilistic	3.1 [2.4-4]	[1.4-6.9]	21	1222	18624	1.4 [1.1-1.8]	49.4 [16.3-69.5]	0.006	0.232	
Probabilistic	2 [0.6-7.1]	NA	1	17	25	NA	NA	1	NA	
**Timing of exposure collection**										0.99
Prospetively	3 [2.4-3.9]	[1.7-5.5]	18	1019	18382	1.3 [1-1.7]	40 [0-65.8]	0.041	0.552	
Retrospectively	3.1 [1.5-6.1]	[0.2-46.8]	4	220	267	1.9 [1.1-3.2]	72.6 [22.6-90.3]	0.012	0.387	
**UNSD Region**										0.023
Eastern Europe	1.3 [0.8-2.3]	NA	1	95	113	NA	NA	1	NA	
Northern Europe	3.1 [2.3-4.1]	[1.5-6.3]	15	830	17865	1.3 [1-1.8]	43 [0-69]	0.039	0.249	
Oceania	2.8 [0.8-10]	NA	1	20	20	NA	NA	1	NA	
South America	0.6 [0.1-5.1]	NA	1	14	5	NA	NA	1	NA	
Southern Europe	6 [2.6-14.3]	NA	2	55	60	1	0	0.38	NA	
Western Asia	5.4 [2.3-12.5]	NA	1	70	70	NA	NA	1	NA	
Western Europe	3.1 [1.9-5]	NA	1	155	516	NA	NA	1	NA	
**Age range at recrutment**										0.113
< 6 months	2.8 [2.1-3.9]	[1.7-4.8]	5	369	765	1 [1-1]	0 [0-2.6]	0.931	0.161	
< 1 year	3.3 [2.4-4.6]	[1.3-8.5]	13	668	17695	1.4 [1-2]	51.8 [9.2-74.4]	0.015	0.546	
< 2 years	1.8 [1.1-2.9]	[0.1-53.6]	3	167	154	1.3 [1-2.4]	40.8 [0-81.9]	0.185	0.292	
**Age range at interview**										0.053
2-5 years	4.1 [3-5.6]	[2.9-5.9]	9	403	10168	1 [1-1.3]	0 [0-38.1]	0.804	0.617	
5-10 years	2.3 [1.5-3.6]	[0.8-7.3]	8	579	8160	1.7 [1.2-2.5]	64.8 [24.9-83.5]	0.006	0.472	
10-15 years	2.2 [1-5.1]	NA	1	47	93	NA	NA	1	NA	
15-20 years	6.8 [2.6-17.2]	NA	1	46	92	NA	NA	1	NA	

	OR (95%CI)	95% Prediction interval	N Studies	N LRTI cases	N controls	H (95%CI)	I² (95%CI)	P heterogeneity	P-value Egger test	P-value subgroup difference
**Hospitalization LRTI -**										0.478
Ambulatory	2.9 [2.1-4.1]	[1.2-6.9]	8	656	17954	1.4 [1-2.1]	50.4 [0-77.8]	0.049	0.616	
Hospitalized	4.2 [2.7-6.5]	[2.3-7.4]	7	234	216	1 [1-1.5]	0 [0-54.2]	0.701	0.249	
Hospitalized/ambulatory	3.3 [1.9-5.6]	NA	2	94	186	1	0	0.812	NA	
**Virus screened for LRTI +**										0.249
Adenovirus type 7	2.8 [0.8-10]	NA	1	20	20	NA	NA	1	NA	
Human Metapneumovirus (hMPV)	10 [2.4-41.2]	NA	1	23	30	NA	NA	1	NA	
Human Respiratory Syncytial Virus	3 [2.3-3.8]	[1.3-6.5]	20	1196	18599	1.4 [1.1-1.8]	48.5 [13.6-69.4]	0.008	0.343	
**Type of LRTI**										0.007
Bronchiolitis	3.8 [3-4.7]	[2.9-4.8]	15	772	18214	1.2 [1-1.6]	26.9 [0-60.6]	0.159	0.705	
LRTI not specified	1.9 [1.4-2.7]	[0.9-4.1]	6	447	415	1.2 [1-1.9]	34 [0-73.5]	0.181	0.442	
Pneumonia	2.8 [0.8-10]	NA	1	20	20	NA	NA	1	NA	
**Type of wheezing**										0.996
Any wheezing	3.3 [1.9-5.6]	NA	2	94	186	1	0	0.812	NA	
Current wheezing	3.1 [1.6-6.3]	[0.4-22.8]	6	359	17164	1.9 [1.3-2.9]	72.3 [36-88]	0.003	0.389	
Recurrent wheezing	3 [2.2-4.2]	[1.3-7.2]	13	631	783	1.3 [1-1.8]	44 [0-70.7]	0.045	0.678	
Wheezing in the last 12 months	3.1 [1.9-5]	NA	1	155	516	NA	NA	1	NA	
